# An improved Newman fast division algorithm based on multi-factor correlation for dynamic traffic sub-region control

**DOI:** 10.1371/journal.pone.0343245

**Published:** 2026-03-12

**Authors:** Xiujuan Tian, Jinyong Ding, Huanying Liu, Haoke Deng

**Affiliations:** 1 School of Transportation Science and Engineering, Jilin Jianzhu University, Changchun, Jilin, China; 2 FAW Logistics Co., Ltd, Changchun, Jilin, China; 3 School of Electronics and Information Engineering‌, Ningbo University of Technology‌, Ningbo, Zhejiang, China; Guilin University of Electronic Technology, CHINA

## Abstract

Given the ongoing expansion of urban traffic signal control coverage, it is imperative to systematically delineate sub-regions based on the topology of road networks and traffic flow characteristics to enhance the stability of traffic management systems. To enhance the rationality of dynamic control sub-region division, this paper establishes a traffic-mechanism-driven edge weight construction framework integrated with modilarity-based community detection. This model integrates traffic volume, signal cycle, and traffic density to compute a comprehensive correlation degree between adjacent intersections. Furthermore, an entropy-weighted TOPSIS framework is proposed to objectively evaluate the proximity of neighboring nodes. This proximity measure is subsequently utilized as the edge weight within an improved Newman fast partitioning algorithm, thereby augmenting modularity and guiding the delineation of sub-regions. Empirical results indicate that while the conventional Newman algorithm exhibits limited adaptability to complex traffic patterns, the proposed model yields more distinct and precise sub-region divisions that more accurately reflect actual traffic conditions. Consequently, this methodology provides a robust foundation for the development of more effective signal control strategies.

## 1 Introduction

In response to the persistent process of urbanization, traffic congestion has become increasingly prevalent. The continual expansion of roadway infrastructure, coupled with the sustained rise in vehicle numbers, necessitates the implementation of regionally coordinated traffic control strategies. However, traditional global unified signal control exhibits limitations when applied to contemporary traffic networks. Partitioning signal control into smaller sub-regions can significantly enhance traffic management effectiveness. The concept of partitioning signal control into sub-regions was initially proposed by Walinchus et al. [1]. In circumstances where the network layout is irregular, and traffic patterns within the region exhibit heterogeneity, network decomposition may be justified. Consequently, sub-regions are categorized as either fixed sub-regions or real-time sub-regions.

Generally, signal control sub-regions are classified into static and dynamic categories. While static sub-regions rely on fixed standards or historical data, they often exhibit limited applicability in real-time management. Consequently, current research predominantly emphasizes dynamic sub-regions. Hu Yawen et al. [2] employed traffic data to analyze the interconnection relationships between intersections, thereby reducing delays and enhancing the efficiency of primary roads. Liu Chang et al. [3] introduced a two-layer framework utilizing trajectory data, resulting in more precise and effective clustering. Zhang Man et al. [4] proposed a Dirichlet-based algorithm that redefines density parameters for dynamic segmentation, outperforming traditional static methods. Lin Shu et al. [5] developed a demand balance model aimed at optimizing traffic flow and facilitating real-time delineation of sub-regions.

The correlation among intersections encapsulates their spatial arrangement and traffic dynamics, thereby playing a crucial role in regional coordination and delineation. Key variables such as traffic volume, signal signal cycle, queue lengths, and traffic density substantially influence correlation metrics. For example, Shen et al. [6] utilized fuzzy logic techniques to estimate correlations based on road distance, vehicle flow, and signal cycle, achieving reductions in travel time and stoppages. Bie et al. [7] identified variations in signal cycles, link lengths, and flow discrepancies as critical factors, which informed the development of a robust correlation model for regional delineation. Yagoda et al. [8] proposed real-time correlation computation methods to synchronize traffic signal phases and dynamically adjust boundary offsets. Zhou et al. [9] introduced a rapid regional partitioning approach employing modular optimization, demonstrating empirical efficacy. Wang et al. [10] proposed an adaptive framework incorporating a correlation index (CI), which exhibited strong performance in regional division tasks. Zhai et al. [11] calculated path flows and correlation degrees based on predefined thresholds, effectively reducing network delays.

Researchers have developed advanced algorithms for regional road network segmentation based on graph theory principles. Shiling Li et al. [12] used the Louvain algorithm for more accurate sub-region division. Duanyang Liu et al. [13] combined Pearson correlation and normalization to enhance partitioning with the snake algorithm. Yan Xing et al. [14] integrated a boundary index model with the enhanced Newman algorithm and spectral clustering, improving metrics like delay and stop count. Jing Luo et al. [15] proposed a dynamic partitioning method merging static division with traffic flow density, validated through modeling. Lu Zhang et al. [16] introduced a dynamic control sub-region division based on an improved Label Propagation Algorithm, reducing delays and travel times. pan et al. [17] applied k-means clustering and the METIS algorithm for data-driven traffic segmentation. Fei Yan et al. [18] partitioned traffic networks into sub-regions using coherence measures, α-Cut, and Moran's I, capturing spatiotemporal variations. Xiaohui Lin et al. [19] used Canopy-K-Means for real-time segmentation with high performance. Xiujuan Tian et al. [20] employed node importance and an improved Newman algorithm for better segmentation.

Research primarily differentiates between static and dynamic segmentation techniques. Static methods employ fixed criteria but exhibit reduced effectiveness in scenarios characterized by rapidly fluctuating traffic conditions. Consequently, advanced approaches such as trajectory refinement, density peak analysis, or the solution of Dirichlet problems are employed. However, challenges persist, including dependence on a limited set of similarity factors, reliance on manually set thresholds, and inadequate modeling of complex roadway networks. Graph-based methodologies, notably Louvain and spectral clustering algorithms, encounter difficulties related to subjective weighting schemes, insufficient integration of multiple influencing factors, and suboptimal modeling of node importance. These limitations contribute to boundary instability and impede application in real-time traffic management.

Addressing the aforementioned limitations, this research focuses on the construction of edge weights driven by traffic mechanisms and their integration into a modularity-based Newman fast partitioning algorithm for dynamic sub-region management. The proposed correlation model integrates multi-dimensional features from adjacent intersections, effectively eliminating the reliance on simplistic single-factor similarity measures. An entropy-weighted TOPSIS framework is employed to objectively determine weights and evaluate proximity across multiple factors, thus reducing dependence on manual threshold calibration and subjective weighting schemes. Additionally, the incorporation of node importance into the construction of edge weights and the optimization process significantly enhances the algorithm's sensitivity and interpretability concerning topologically critical nodes. The research further examines the computational complexity of the proposed algorithm and validates its effectiveness through comparative analyses against alternative clustering methods, including the improved Louvain, Label Propagation, and spectral clustering algorithms, utilizing real-world road network data. Evaluation metrics encompass computational efficiency, stability, robustness to noise, and control effectiveness, exemplified by measures such as delay reduction and minimization of stoppages.

The core innovation of this paper lies in the traffic-mechanism-driven edge weight construction integrated with modularity-based community detection, with the primary contributions summarized as follows:

(1) This paper proposes an enhanced correlation model that integrates multi-source traffic features, employing entropy-weighted TOPSIS for objective weighting of factors and proximity analysis to mitigate the subjectivity associated with manual threshold settings.
(2) The Newman fast partitioning framework strategically modifies edge weights based on node significance and incorporates a module optimization mechanism. It employs an enhanced algorithm aimed at increasing accuracy and protecting essential nodes.
(3) The quality of partitioning, enhancements in traffic management, and overall efficiency of the proposed method were empirically validated using real-world case studies. Additionally, its robustness and adaptability were evidenced through comprehensive ablation analyses.

The following section explains the construction and implementation of the improved correlation model and the Newman algorithm, compares and evaluates the method's performance using simulations and real-world cases, and discusses limitations and deployment recommendations.

## 2 Research on the improved correlation degree model

**Fig 1:**
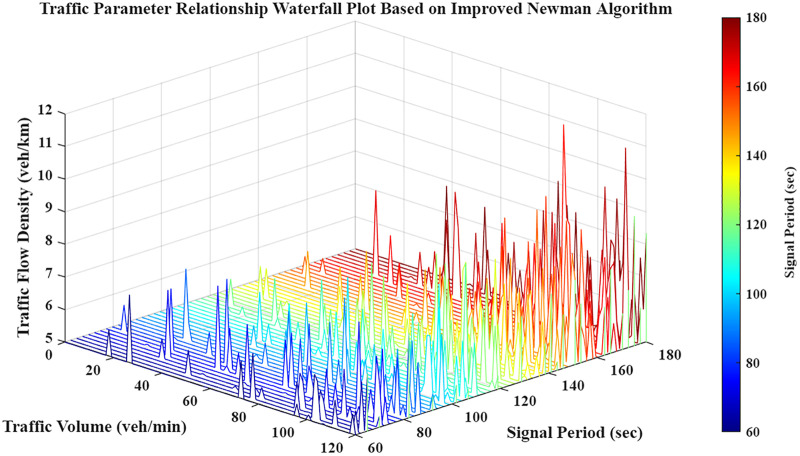
Waterfall chart of traffic correlation parameter relationships.

This study quantifies the interaction strength between adjacent intersections by integrating invariant spatial distance with dynamic traffic parameters: volume, signal cycle, and density. These factors establish a logical framework for sub-region partitioning. As illustrated in Fig 1, these connectivity parameters fluctuate according to real-time traffic states. Equation (3) calculates the aggregate correlation using a weighted geometric mean, incorporating normalization and lower-bound smoothing to ensure numerical stability across varying conditions.

To ensure conceptual clarity, this study establishes a hierarchical transformation pipeline for the core metrics. The Correlation Degree ($I_{ij}$) quantifies the interaction strength between adjacent intersections derived from individual traffic factors, including volume, signal cycle, and density. These multi-dimensional factors are subsequently fused into the Relative Closeness ($D_{i}$) through an entropy-weighted TOPSIS framework, providing an objective evaluation of node pairs relative to an ideal coordination state. This comprehensive index is ultimately formulated as the Edge Weight ($w_{ij}$) through a symmetric function, serving as the primary graph-theoretic input for the improved Newman algorithm to optimize modularity and delineate dynamic traffic sub-regions.

This hierarchical structure ensures that this methodology objectively transforms raw traffic dynamics into stable structural weights, facilitating the integration of traffic mechanisms into modularity-based network partitioning.

### 2.1 Construction of comprehensive correlation calculation model

Step 1. Perform min–max normalization on each raw indicator $x_{i}$, refer to Equation (1).


$$I_{ij}^{\left(k\right)}=\frac{X_{ij}^{\left(k\right)}-min\left(X^{\left(k\right)}\right)}{max\left(X^{\left(k\right)}\right)-min\left(X^{\left(k\right)}\right)}\text{\textbackslash{}hspace\{1em}\}(k=1,2,3)$$ (1)

where $I_{i,j}^{\left(k\right)}$ denotes the normalized correlation for the k-th feature (traffic volume, signal cycle, or density), with values closer to 1 indicating stronger correlation. The terms $max\left(X^{\left(k\right)}\right)$ and $min\left(X^{\left(k\right)}\right)$ represent the maximum and minimum values of the k-th indicator within the time window, respectively.

Step 2. To safeguard against the overall correlation approaching zero in instances where a subterm is exactly zero during multiplication, and to improve numerical stability and robustness to measurement noise, we introduce a lower truncation (smoothing term). Selecting the smoothing parameter ε is vital for model fidelity. Values exceeding 0.15 diminish discriminative power, while those below 0.01 compromise numerical stability. Adopting ε=0.05 balances sensitivity to traffic variations with mathematical robustness, refer to Equation (2).


$$I_{ij}^{\prime \left(k\right)}=max\left(I_{ij}^{\left(k\right)},\varepsilon \right)$$ (2)

where $I_{i,j}^{\prime \left(k\right)}$ represents the truncated sub-association degree of category k, with a range of [ε,1]. ε denotes the smoothing lower bound (safety buffer), to avoid numerical instability caused by weights approaching zero, a smoothing term [21] is introduced, with a value range of $\varepsilon \in \left[10^{-2},5\times 10^{-2}\right]$.

Step 3. Construction of the comprehensive cascade correlation model.


$$I_{ij}=\alpha {I^{\prime }}_{q\left(ij\right)}⬝\beta {I^{\prime }}_{C\left(ij\right)}⬝c{I^{\prime }}_{\rho \left(ij\right)}$$ (3)

where $I_{q\left(ij\right)}$ is the intersection i and j the degree of traffic correlation between them. For intersections, the signal cycle correlation degree between them. $I_{\rho \left(ij\right)}$ is the correlation degree of traffic flow density between intersections i, j. α, β, c is the correlation degree weight coefficients are respectively, and their values are determined by the entropy weight method [22]. The method allocates feedback weights based on the index's information entropy. Specifically, lower values of information entropy correspond to higher feedback weights, whereas higher values of information entropy are associated with reduced weights.

Equation (3) employs a multiplicative model to capture the bottleneck effect across traffic demand $I_{q\left(ij\right)}$, signal cycle feasibility $I_{C\left(ij\right)}$, and spatial connectivity $I_{\rho \left(ij\right)}$. Unlike additive frameworks where high volume may mask cycle incompatibility, this research utilizes a product approach to ensure aggregate correlation $I_{ij}$ is dictated by the weakest functional link. This logic provides a rigorous filter for sub-region aggregation by excluding intersections with fundamentally incompatible operational states.

In systems engineering, a product relationship accurately measures the effectiveness of such serial structures. Unlike a weighted sum, this model ensures that if any critical factor approaches zero—for instance, due to insufficient traffic volume, mismatched signal cycles, or sparse vehicle density—the overall coordination benefit nullifies. This captures the cascading effect where the performance of the interconnected micro-system is limited by its weakest component. Consequently, intersections lacking any of these essential correlations are correctly excluded from the same sub-region.

**Fig 2:**
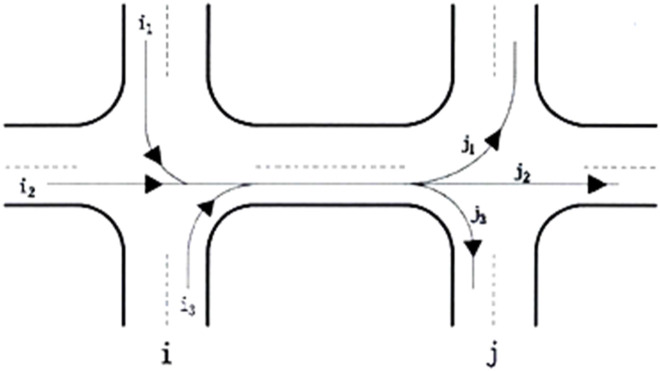
Correlation between traffic flow direction and volume at adjacent intersections.


The diagram depicting the two-way roadway segment between successive intersections. In cases where one direction of the two-way segment necessitates coordination and control, it is imperative that both directions are subjected to coordination and control measures. Therefore, the two-way road section system can be divided into two subsystems, denoted as $I_{ij}$ and $I_{ji}$, as shown in Fig 2. The road section correlation from intersection i to j is $I_{ij}$, and from j to i is $I_{ji}$. The overall correlation degree is taken as the maximum correlation degree between the two subsystems, as shown in Equation (4).

$$I_{ij}=I_{ji}=max\left(I_{i\to j},I_{j\to i}\right)$$ (4)

### 2.2 Traffic correlation degree

Traffic flow is inherently linked to intersection correlation. An increase in traffic volume reduces available driving space and prolongs queue lengths downstream, thereby exerting a negative influence on upstream conditions. The Whitson model, as outlined in the American Traffic Control System manual [23], quantitatively assesses this correlation, as represented in Equation (5).


$$I=\frac{0.5}{1+t}\left[\frac{nq_{\text{max}}}{\sum_{j=1}^{n}q_{j}}-1\right]$$ (5)

Here, t represents the travel time between intersections (in minutes). $q_{\text{max}}$ defines the maximum traffic volume (vehicles per hour) at the upstream intersection i, i including $i_{\text{1}}$, $i_{\text{2}}$, $i_{\text{3}}$. $\sum_{i=1}q_{i}$ is the traffic volume of the lane group of the entrance lane at the downstream intersection j (vehicles per hour). n represents the number of associated flow directions at the upstream intersection i of the entrance lane of the intersection.

Accurately assessing the correlation between adjacent intersections requires accounting for the discrete nature of traffic flow as it propagates downstream. While the standard Whitson model characterizes the relationship between correlation degree, inter-intersection distance, and flow rate, it lacks an explicit mechanism for flow discreteness. To bridge this gap, we enhance the Whitson model by integrating a queue dispersion model and the interconnection expectation index [24]. Furthermore, as formulated in Equation (6), the temporal variable is redefined as either the travel duration from the upstream intersection to the downstream queue tail or the mean travel time under free-flow conditions at the entry point.


$$I_{q\left(i↔j\right)}=\frac{\text{1}}{n-1}\left(\frac{n\mathrm{\backslash stackrel}i↔jq_{\text{max}}}{\sum_{i=1}^{n}\mathrm{\backslash stackrel}i↔jq_{i}}-1\right)\frac{\text{1}}{1+t}$$ (6)

where $I_{q\left(i↔j\right)}$ represents the flow correlation degree between intersections i and j. n is the number of inflow and flow directions at the upstream intersection. $\mathrm{\backslash stackrel}i↔jq_{\text{max}}$represents the maximum traffic volume (vehicles per hour) at the upstream intersection i, and i includes $i_{\text{1}}$, $i_{\text{2}}$, $i_{\text{3}}$. $\sum_{i=1}\mathrm{\backslash stackrel}i↔jq_{i}$ represents the traffic volume (vehicles per hour) of the lane group at the j entrance of the downstream intersection. t represents the time from the upstream intersection to the end of the queue at the downstream intersection or the average driving time (in minutes) when no vehicle queues at the entrance. The calculation of time t is as shown in Equation (7).


$$t=\frac{L-l}{\overset{―}{V}}$$ (7)

where L is the distance (m) between adjacent intersections. l represents the average queue length (m) at the downstream intersection. $\overset{―}{V}$ represents the average speed of traffic flow on the road section.

To account for the bidirectional nature of traffic between adjacent intersections, the correlation degree must incorporate both flow directions. As formulated in Equation (8), we select the maximum correlation value between the two directions to represent the definitive coupling strength between neighboring intersections.


$$I_{q\left(i,j\right)}=\text{MAX}\left({I^{\text{1}}}_{q\left(i,j\right)},{I^{\text{2}}}_{q\left(j,i\right)}\right)$$ (8)

where ${I^{\text{1}}}_{q\left(i,j\right)}$ represents the traffic flow direction from the upstream intersection i to the downstream intersection j.${I^{\text{2}}}_{q\left(j,i\right)}$ represents the traffic flow direction from downstream intersection j to upstream intersection i.

**Fig 3:**
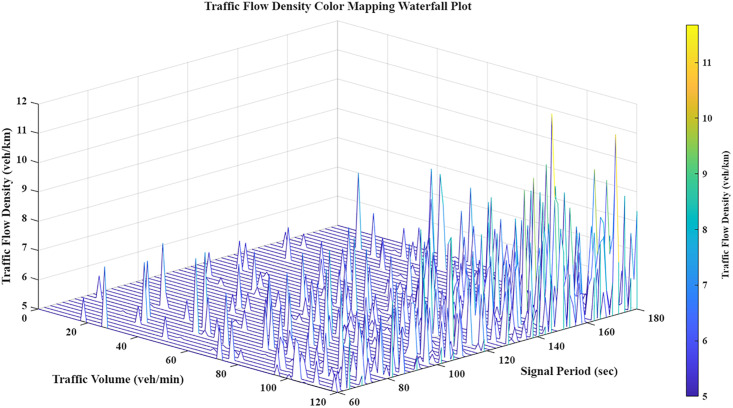
Traffic volume waterfall chart.

Traffic volume is a primary determinant of accuracy in both grey correlation and probabilistic graphical models. Fig 3 illustrates the 3D distribution of these parameter interrelations, providing the quantitative and visual evidence necessary to substantiate the model analysis.

### 2.3 Signal period correlation degree

Signal cycle disparities between adjacent nodes significantly impact network connectivity. To maintain stable phase relationships, sub-region nodes should be synchronized with the primary node or the regional maximum cycle length. Although Webster’s formula suggests that extending cycles enhances capacity, it simultaneously increases delays; therefore, uniform cycles are essential for stabilizing phase differences and optimizing coordinated control. These variations are critical parameters when delineating sub-divisions. The relationship between intersection correlation and the period duration factor [25] is formalized in Equation (9), which incorporates cycle length as a fundamental variable.


$$I_{C\left(i,j\right)}=2\times \left[1.5-\frac{max\left(C_{i},C_{j}\right)}{min\left(C_{i},C_{j}\right)}\right]$$ (9)

where $I_{C\left(i,j\right)}$ represents the correlation degree of the signal cycle duration.$C_{i}$and $C_{j}$ are the signal cycle durations of intersectioniand intersection j respectively. $max\left(C_{i},C_{j}\right)$ is the maximum of the signal cycle durations of the two. $min\left(C_{i},C_{j}\right)$ is the one with the smallest signal cycle duration between the two.

**Fig 4:**
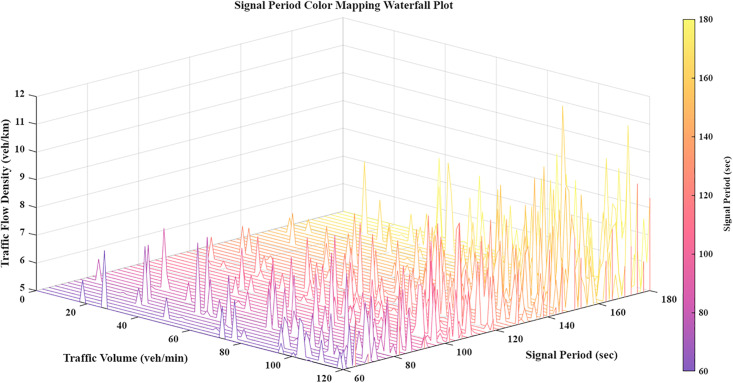
Signal cycle mapping waterfall chart.

As a fundamental parameter, the signal cycle facilitates both correlation modeling and sub-region delineation. It provides the essential input for regional coordinated control, directly influencing the roadway network's operational efficiency. Fig 4 presents a waterfall chart mapping these signal cycle dynamics, illustrating its behavior across spatial and temporal dimensions.

### 2.4 Correlation degree of traffic flow density

Sub-region delineation requires an integrated analysis of traffic flow, signal cycle duration, and road segment density. As a primary indicator of congestion, segment density determines the necessity for signal coordination between adjacent intersections. The correlation degree relative to this density [26] is quantified through Equations 10‒11.


$$I_{\rho \left(i↔j\right)}=min\left(max\left(\frac{\rho_{\text{i}\to \text{j}}}{\rho_{\text{s i}\to \text{j}}},\frac{\rho_{\text{j}\to \text{i}}}{\rho_{\text{s j}\to \text{i}}}\right),1\right)$$ (10)

$$\rho_{\text{i}\to \text{j}}=\frac{q_{\text{i}\to \text{j}}}{n_{\text{i}\to \text{j}}L_{\text{i}\to \text{j}}}$$ (11)

where $I_{\rho \left(i↔j\right)}$ represents the density correlation degree between intersections iand j. $\rho_{\text{i}\to \text{j}}$ represents the traffic flow density of the section from intersection i toj. $\rho_{\text{s i}\to \text{j}}$ represents the traffic flow density of the section from intersection i to intersection j in the saturated state. $q_{\text{i}\to \text{j}}$ represents the total flow from intersection i to intersection j within a specific period of time. $n_{\text{i}\to \text{j}}$ represents the number of lanes from intersection i to intersection j. $L_{\text{i}\to \text{j}}$ represents the length (m) of the section of road between adjacent intersections i and j.

**Fig 5:**
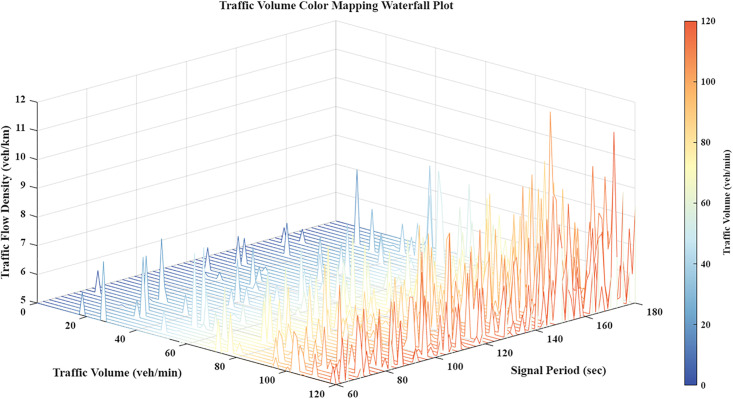
Traffic flow density waterfall chart.

Traffic flow density is a fundamental parameter for quantifying road space utilization, saturation, and the operational efficiency of segments between intersections. To analyze the interrelationships among density, volume, and cycle duration, we developed the mapping presented in Fig 5. This waterfall chart provides a precise visualization of the underlying spatiotemporal correlation dynamics within the model.

## 3 Construction of edge weights via entropy–TOPSIS correlation model

The preceding section analyzed the correlation levels between intersections utilizing variables such as traffic volume, signal cycle, and density. This section introduces the entropy weight-TOPSIS methodology to establish a composite index for graph-based partitioning. The model computes a closeness measure functioning as an edge weight, thereby facilitating the enhanced application of the Newman fast division algorithm.

**Fig 6:**
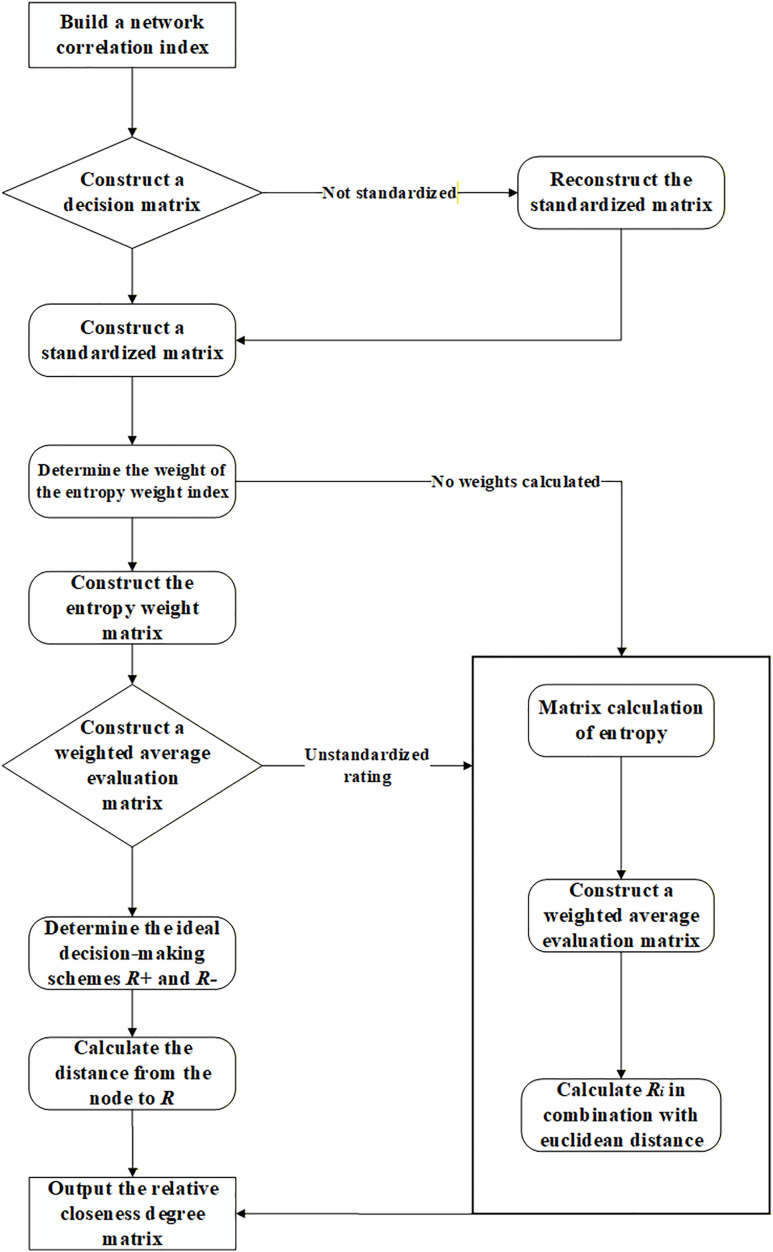
Flowchart of the entropy weight-based TOPSIS algorithm model.

Nodes constitute fundamental components within the network architecture, with their interconnections fundamentally influencing the network's efficiency and structural configuration. The ranking of node importance facilitates the identification of pivotal nodes through methodologies such as PageRank [27], NI-LPA [28], and importance matrices [29]. These algorithms, which depend on topological properties, face limitations when applied to real-time data and are often incapable of managing high-frequency dynamism. In transport networks, the strength of the links (edges) is of paramount importance. This research endeavors to develop dynamic, real-time edge weights that precisely mirror traffic conditions, addressing the inflexibility inherent in traditional static methods. The study utilizes the entropy weight-based TOPSIS algorithm, incorporating multi-dimensional traffic data to enable real-time updates. Fig. 6 depicts the model workflow.

### 3.1 Edge weight determination in complex networks: A multidimensional correlation entropy-weighted TOPSIS model

This research utilizes the entropy-weighted TOPSIS methodology not only as a comprehensive evaluation framework but also specifically to ascertain edge weights between adjacent intersections, quantifying their interrelations. By incorporating traffic flow, signal cycle, and traffic density as evaluation metrics, the approach applies objective weighting procedures and calculates the relative closeness to illustrate the mutual influence of traffic conditions across different intersections through edge weights. This procedure establishes a foundation for subsequent correlation analyses and network modeling. The principal objective of the model is to compute a relative closeness metric, which functions as the weight of the edge linking two intersections (nodes), providing critical input for the subsequent application of the refined Newman fast division algorithm.

The methodology for determining edge weights utilizing the entropy weight-based TOPSIS model is delineated as follows.

1) Step 1. Construct the evaluation matrix. Using adjacent intersections as samples, a decision matrix $X=[x_{ij}\;{]}_{m\times n}\;$ is built, where m represents the number of samples and n=3 (corresponding to the correlation degrees of traffic flow, signal cycle, and traffic density). All three indicators are viewed as positive attributes, meaning higher values indicate stronger correlations. Construct decision matrix values as shown in Table 1.

**Table 1:** Decision matrix values.

i−j	traffic volume correlation degree	signal cycle correlation degree	traffic density correlation degree
1-2	0.566431094	0.44160038	0.365800983
1-6	0.910140974	0.943438167	0.851181545
2-3	0.743433588	0.659714192	0.606046976
2-7	1.500490935	1.485614801	1.5977249
3-4	0.876625564	0.755942548	0.848160061
3-9	0.594558076	0.437124828	0.454446769
4-5	0.862274218	0.857203023	0.888388964
4-10	1.166353273	1.132085742	1.09446845
5-11	0.912035463	0.798375539	0.806193717
6-7	0.731655178	0.680412821	0.855179908
7-8	0.429224157	0.594829286	0.584318701
7-18	0.958862086	0.951838153	0.849194692
8-9	0.310023738	0.459768572	0.50656592
8-17	0.880541703	0.912916764	0.769560301
9-10	1.043894124	1.074404521	1.327660493
9-16	0.590940524	0.625850676	0.754219634
10-11	1.415564282	1.446759981	1.272670467
10-15	0.980706262	0.859156732	0.855371555
11-12	0.459441984	0.430311479	0.432270493
12-13	0.446328997	0.428057365	0.363655186
13-14	0.45836343	0.451241152	0.454178539
14-15	0.635550416	0.631765391	0.458147618
15-16	0.900583647	0.730542498	0.65782378
16-17	0.514885297	0.398039428	0.584154265
17-18	0.446447133	0.510623601	0.205624925


$$X=(x_{ij}{)}_{\text{m}\times \text{n}}(i=1,2,\cdots ,m;j=1,2,\cdots ,n)\;$$ (12)

$$\begin{array}{c} X={\left(\begin{array}{cccc} @cccc@{\text{x}}_{11} & {\text{x}}_{12} & \cdots & {\text{x}}_{1n}\\ {\text{x}}_{21} & {\text{x}}_{22} & \cdots & {\text{x}}_{2n}\\ \vdots & \vdots & \ddots & \vdots \\ {\text{x}}_{m1} & {\text{x}}_{m2} & {\text{x}}_{m3} & {\text{x}}_{mn} \end{array}\right)}_{m\times n} \end{array}$$ (13)

where $x_{ij}$ denotes the performance value of the i-th alternative with respect to the j-th criterion. The matrix functions as the foundational dataset for the model, with each row representing a pair of neighboring intersections and each column denoting a correlation indicator. The first column indicates traffic volume correlation $I_{q}$, the second column indicates signal cycle correlation $I_{c}$, and the third column reflects traffic density correlation $I_{k}$.

2) Step 2. Construction of a standardized matrix. Standardization procedures are implemented to ensure data comparability and to differentiate between positive and negative indicators. The matrix undergoes standardization, and subsequent processing involves the application of equations such as (14) and (15).

$$y_{ij}=\frac{x_{ij}}{\sqrt{\sum_{i=1}^{\text{m}}x_{ij}^{\text{2}}}},\text{\textbackslash{}hspace\{1em}\}(i=1,2,\cdots ,m;j=1,2,\cdots \;,\text{n)}$$ (14)

$$\begin{array}{c} Y={\left[\begin{array}{cccc} @cccc@y_{11} & y_{12} & \cdots & y_{1n}\\ y_{21} & y_{22} & \cdots & y_{2n}\\ \vdots & \vdots & \ddots & \vdots \\ y_{m1} & y_{m2} & \cdots & y_{mn} \end{array}\right]}_{m\times n} \end{array}$$ (15)

where Y is the standard matrix in the equation. $y_{ij}$ is the parameter in the i-th row and j-th column of the standard matrix. $\sum_{i=1}^{\text{m}}x_{ij}^{\text{2}}$ represents the process of normalizing the parameters of the i-th row and j-th column of the evaluation matrix is carried out, and the normalized data $y_{ij}$ is brought into the entropy weight method. Given that the three correlation indicators may differ in scale and magnitude—such as traffic volume, which is generally higher than density—this phase involves the standardization process to eliminate dimensional effects and facilitate comparability among indicators. This procedure ensures that, in subsequent comprehensive assessments, no single indicator exerts disproportionate influence on the results due to its raw numerical value, thereby promoting a more balanced evaluation.

3) Step 3. Determination of the entropy index weight. Initially, the feature proportion pertaining to the objective function is computed. Consistent with the principles of entropy [30–32], an increase in informational content corresponds to a reduction in entropy value. Accordingly, the entropy weight serves as an indicator of the amount of information conveyed. The relevant calculation formula is presented in Equation (16).

$$p_{ij}=\frac{y_{ij}}{\sum_{i=1}^{m}y_{ij}}$$ (16)

where $p_{ij}$ represents the feature proportion under the i-th row and the j-th column. $y_{ij}$ is the parameter in the i-th row and j-th column of the standard matrix.

Secondly, calculate the entropy value $e_{j}$ under the j indicator. The calculation formula is as shown in Equation (17).


$$e_{j}=-\frac{\text{1}}{lnm}\sum_{i=1}^{m}P_{ij}\;ln\;P_{ij},\text{\textbackslash{}hspace\{1em}\}e_{j}\in \left[0,1\right]$$ (17)

Here, $e_{j}$ represents the information entropy value. $p_{ij}$ represents the feature proportion under the i-th row and the j-th column.

Finally, calculate the weights and the calculation formula, such as equations (18)~(20).


$$w_{j}=\frac{1-e_{j}}{\sum_{j=1}^{m}(1-e_{j})}$$ (18)

$$W={\left(w_{j}\right)}_{m\times n}$$ (19)

$$\begin{array}{c} W={\left(\begin{array}{cccc} @cccc@w_{11} & w_{12} & \cdots & w_{1n}\\ w_{21} & w_{22} & \cdots & w_{2j}\\ \vdots & \vdots & \ddots & \vdots \\ w_{m1} & w_{m2} & \cdots & w_{mn} \end{array}\right)}_{m\times n} \end{array}$$ (20)

where $e_{j}$ is the information entropy value in the formula. $w_{j}$ is the weight, $w_{j}\in \left[0,1\right]$. W is the entropy weight matrix.

The entropy weight method objectively determines indicator weights by quantifying dispersion via entropy, which reflects the amount of information conveyed by an indicator. Indicators exhibiting high variability, such as traffic volume, possess low entropy and consequently are assigned higher weights, whereas more stable indicators, like signal signal cycle, have higher entropy and thus lower weights. For example, when traffic volume demonstrates significant variation across intersections while signal cycles remain relatively constant, the method assigns a greater weight to “traffic volume” due to its superior capacity to differentiate among intersections. The entropy weight table is presented in Table 2.

**Table 2:** Entropy weight distribution table.

Indicator Name	Entropy Value $\;e_{j}$	Information Utility Value	Weight $\;w_{j}$	Weight Percentage
Traffic Volume Correlation	0.930936244	0.069063756	0.283456255	28.3456255
Signal Cycle Correlation	0.884362242	0.115637758	0.474608505	47.4608505
Traffic Density Correlation	0.941052787	0.058947213	0.24193524	24.193524

4) Step 4. Construction of the Weighted Average Evaluation Matrix. The standardized evaluation matrix, in conjunction with the entropy weight matrix derived via the entropy weight method, is employed to assign weights to the matrix, thereby computing the weighted evaluation matrix. The corresponding calculation procedures are detailed in Equations 21~22.

$$Z={\left(z_{ij}\right)}_{m\times n},\text{\textbackslash{}hspace\{1em}\}z_{ij}=y_{ij}\cdot w_{j}$$ (21)

$$\begin{array}{c} Z={\left(\begin{array}{cccc} @cccc@z_{11} & z_{12} & \cdots & z_{1n}\\ z_{21} & z_{22} & \cdots & z_{2n}\\ \vdots & \vdots & \ddots & \vdots \\ z_{m1} & z_{m2} & \cdots & z_{mn} \end{array}\right)}_{\;\;m\times n} \end{array}$$ (22)

where Z in the formula is the weighted evaluation matrix. $z_{ij}$ is the parameter of the i-th row and j-th column of the weighted matrix. $y_{ij}$ is the parameter in the i-th row and j-th column of the standard matrix. $w_{j}$ is the weight, $w_{j}\in \left[0,1\right]$.This step combines the standardized data with objective weights. Each element $\;z_{ij}$ in matrix 𝐙, this metric denotes the weighted normalized correlation value, reflecting both the positional relationship of the intersection relative to a specific indicator and the indicator's significance within the comprehensive evaluation of the current road network.

5) Step 5. Calculate the weighted standardized ratings to determine the positive and negative ideal decision schemes $R^{+}$and $R^{-}$respectively. The calculation formulas are as shown in equations (23)~(24).

$$R^{+}=\left\{\underset{i\in L}{max}(z_{i1},z_{i2},\ldots ,z_{in})\right\}=\left\{z_{\text{1}}^{max},z_{\text{2}}^{max},\ldots ,z_{n}^{max}\right\}$$ (23)

$$R^{-}=\left\{\underset{i\in L}{min}(z_{i1},z_{i2},\ldots ,z_{in})\right\}=\left\{z_{\text{1}}^{min},z_{\text{2}}^{min},\ldots ,z_{n}^{min}\right\}$$ (24)

where $R^{+}$ represents a positive ideal decision scheme in the formula. $R^{-}$ represents a negative ideal decision scheme. L={1,2,…,m}. The positive ideal solution $R^{+}$ represents an ideally optimal adjacent intersection pair that achieves the best values across all three correlation indicators, unlike the negative ideal solution. $R^{-}$ corresponds to an ideal worst-case adjacent intersection pair with the lowest values in all three indicators.

6) Step 6. The Euclidean distance is employed to quantify the separation between each node and the positive and negative ideal solutions. This methodological approach facilitates the calculation of the closeness coefficient. The corresponding mathematical expressions are delineated in equations (25)~(27) .

$$R_{i}^{+}={\left[\sum_{j=1}^{m}(z_{ij}-z_{j}^{max}{)}^{\text{2}}\right]}^{1/2}$$ (25)

$$R_{i}^{-}={\left[\sum_{j=1}^{m}(z_{ij}-z_{j}^{min}{)}^{\text{2}}\right]}^{1/2}$$ (26)

$$D_{i}=\frac{R_{i}^{-}}{R_{i}^{+}+R_{i}^{-}}$$ (27)

where $D_{i}$ represents the relative closeness of node i, which is defined as the edge weight $w_{ij}$ for the connection between node i and its adjacent nodes. $R_{i}^{+}$ represents the gap between the i-th pair of actual intersections and the ideal intersection pair in terms of comprehensive traffic characteristics. A smaller distance indicates better performance of the intersection pair. $R_{i}^{-}$ denotes the gap between the i-th pair of actual intersections and the worst intersection pair. A larger distance indicates the intersection pair is less unsatisfactory. The relative closeness $D_{i}$ is a value between 0 and 1 indicating how close the intersection pair is to perfection or the worst case. $D_{i}$ is a value near 1 indicates a stronger intersection correlation and closer to the ideal.

7) Step 7. Construct Edge Weights $w_{ij}$. Define the weight $w_{ij}$ of any connecting edge i between two adjacent intersections j as a symmetric function of the relative proximity $D_{i}$ and $D_{j}$ between the two nodes, using the arithmetic mean as follows:

$$w_{ij}\;=\frac{D_{i}\;+D_{j}}{\text{2}}\;$$ (28)

Relative closeness measures how close a scheme is to optimal or worst outcomes [33]. A higher edge weight indicates greater similarity between intersections regarding traffic, signals, and density, reflecting their compatibility. Grouping these in the same control zone with coordinated strategies like green wave synchronization can enhance traffic management. This metric is vital in defining community boundaries in the enhanced Newman algorithm, as shown in Table 3.

**Table 3:** Edge weight value.

i−j	$w_{ij}$	i−j	$w_{ij}$
1-2	0.49	8-17	0.88
1-6	0.89	9-10	1.1
2-3	0.61	9-16	0.63
2-7	1.53	10-11	1.41
3-4	0.89	10-15	0.84
3-9	0.51	11-12	0.47
4-5	0.85	12-13	0.36
4-10	1.06	13-14	0.42
5-11	0.85	14-15	0.58
6-7	0.79	15-16	0.77
7-8	0.6	16-17	0.5
7-18	0.96	17-18	0.38
8-9	0.34		

Relative closeness evaluates the proximity of a configuration to the ideal solution, serving here as the basis for edge weights. These weights quantify the similarity between adjacent intersections by integrating variations in traffic flow, signal cycles, and density. Within our enhanced Newman algorithm, higher edge weights drive the modularity calculation, signaling stronger node connections and a higher probability of sub-region aggregation. By functioning as a dynamic similarity measure, this weighted approach improves partitioning accuracy over static methods by effectively capturing real-time traffic dynamics.

## 4. Dynamic control sub-region division based on the improved Newman fast division algorithm

Utilizing a graph-theoretic abstraction, intersections are represented as nodes, while the connecting road segments between adjacent intersections are depicted as edges within the network. The correlation degree index is incorporated into the TOPSIS model, which employs the entropy weight method to quantify relative closeness. This measure of relative closeness is subsequently used as the edge weight between nodes in an enhanced version of the Newman fast partitioning algorithm. Consequently, this paper articulates an improved sub-region division methodology centered on traffic-mechanism-driven edge weights and modularity optimization.

### 4.1 Newman's Fast Partitioning algorithm

Conventional algorithms such as the Kernighan-Lin (K-L) method [34] and spectral bisection [35] require prior knowledge of the number of communities, which limits their applicability in practical scenarios. The Girvan-Newman algorithm demonstrates high computational complexity, thereby restricting its utility in analyzing complex networks. The Fast Newman (FN) algorithm, developed by Newman et al. [36,37] and based on the Girvan-Newman (GN) approach [38], employs a greedy local search strategy aimed at maximizing modularity, a quantitative metric used to evaluate the quality of a given partition. A higher modularity value signifies a more optimal community division.

The FN methodology assesses variations in the modularity-added value both prior to and subsequent to the formation of network communities. Nodes are successively aggregated based on associated increases in modularity, with the iterative process ceasing once the entire set of nodes has been unified. A dendrogram, generated at the point of peak modularity, depicts the connectivity among nodes and thus delineates the optimal partitioning of sub-regions. The procedural steps of the algorithm are detailed below.

1) Step 1.The preliminary stage entails abstracting the road network into a topological graph. The intersections within the network are quantitatively characterized, and the area is subdivided into m discrete communities, each represented by a node symbolizing a sub-region.
2) Step 2. Construct the m-order symmetric auxiliary matrix E, where element $e_{ij}$ is the proportion of the number of connections between communities i and j to the total number of edges in the network. Establish an array A, whose element $a_{i}$ is the proportion of the number of edges connected between the internal nodes and external nodes of sub-region i to the total number of edges in the network, and the number of elements in array A is equal to the total number of nodes in the network. Where initialize $e_{ij}$ and $a_{i}$ to satisfy equations (29)~(30), and construct the matrix E and array A as equations (31)~(32).

$$\begin{array}{c} e_{ij}=\left\{ \begin{array}{cc} @ll@\frac{\text{1}}{2m}, & \text{Nodes }i\text{ and }j\text{ are connected by edges}\\ 0, & \text{Nodes }i\text{ and }j\text{ are boundlessly connected} \end{array}\right. \end{array}$$ (29)

$$a_{i}=\frac{k_{i}}{2m}$$ (30)

$$\begin{array}{c} A=\left[\begin{array}{cccc} @cccc@a_{\text{1}} & a_{\text{2}} & \cdots & a_{\text{m}} \end{array}\right] \end{array}$$ (31)

$$\begin{array}{c} E=\left[\begin{array}{cccc} @cccc@e_{11} & e_{12} & \cdots & e_{1\text{m}}\\ e_{21} & e_{22} & \cdots & e_{2\text{m}}\\ \vdots & \vdots & \ddots & \vdots \\ e_{\text{m}1} & e_{\text{m}2} & \cdots & e_{\text{mm}} \end{array}\right] \end{array}$$ (32)

where $e_{ij}$ represents the proportion of the number of connections between communities i and j to the total number of edges in the network. $a_{i}$ represents the proportion of the number of edges connected between the internal nodes and external nodes of sub-region ito the total number of edges in the network. $k_{i}$ is the degree of node i, that is, the number of edges connected to node i. m is the total number of edges of the connected nodes in the network.

3) Step 3. Merge two communities according to their similarity or Euclidean distance. The equation used to calculate the modularity increment is presented in Equation (33).

$$\Delta Q=e_{ij}+e_{ji}-2a_{i}a_{j}=2\left(e_{ij}-a_{i}a_{j}\right)$$ (33)

where ΔQ in the formula represents the increment of modularity.

4) Step 4. Repeat the process from Step 2 to Step 3 until the entire network merges into a single sub-region. The algorithm concludes, and no more than one merge is necessary.

The process of aggregating nodes into communities within the network is depicted through a hierarchical tree diagram. The partitioning of this tree, which illustrates the node connection relationships corresponding to the maximum modularity Q, signifies the optimal method for segmenting the network.

### 4.2 Improved Newman fast partitioning algorithm

The Newman fast partitioning algorithm, originally designed for unweighted networks, has been improved for weighted networks by considering the relative closeness degree derived from the correlation degree index input into the TOPSIS algorithm with entropy weights to construct edge weights for dividing sub-regions. Assuming that the network is divided into n communities, denoted as $\left(c_{\text{1}},c_{\text{2}},c_{\text{3}},\cdot \cdot \cdot ,c_{n}\right)$, the symmetric matrix $𝐂=\left[c_{ij}\right]$ is defined, as equation (34), and the modularity calculation formula is redefined, as equation (35).


$$\begin{array}{c} 𝐂=\left[\begin{array}{cccc} @cccc@c_{11} & c_{12} & \cdots & c_{1n}\\ c_{21} & c_{22} & \cdots & c_{2n}\\ \vdots & \vdots & \ddots & \vdots \\ c_{n1} & c_{n2} & \cdots & c_{nn} \end{array}\right]\text{\textbackslash{}hspace\{1em}\}c_{ij}=c_{ji}\text{\textbackslash{}hspace\{0.33em}\}(\forall i,j=1,2,\ldots ,n) \end{array}$$ (34)

$$Q_{w}=\sum_{i}\left(c_{ii}-v_{i}^{\text{2}}\right)=Tre-\|{𝐜}^{\text{2}}\|$$ (35)

where $c_{ij}$ is the sum of the edge weights of communities i and j, indicating the ratio of edge weights within the sub-region to those in the network. $v_{i}=\sum_{j=1}^{n}c_{ij}$ is the sum of the elements in each row of the matrix, representing the ratio of the edge weights of the connections between the internal nodes and external nodes of sub-region i to the network edge weights. Tre is the sum of the elements on the diagonal of matrix C, representing the proportion of edge weights within the sub-region to all edge weights. $\|c^{\text{2}}\|$ is the sum of all the elements of matrix $c^{\text{2}}$.

The improved Newman fast partitioning algorithm considers the correlation degree between network nodes and uses TOPSIS's relative closeness degree, based on entropy weights, as edge weight. Therefore, the elements $e_{ij}$ and $a_{i}$ in matrix E are improved, as shown in equations (36)~(37).


$$\begin{array}{c} e_{ij}=\left\{ \begin{array}{cc} @ll@\frac{D_{ij}}{\sum_{\left(i,j\right)}D_{ij}}, & \text{Node }i\text{ is directly connected to }j\text{ by an edge}\text{;}\\ 0, & \text{Node }i\text{ is directly connected to node }j\text{ without borders}\text{;} \end{array}\right. \end{array}$$ (36)

$$a_{i}=\frac{\sum_{j}e_{ij}}{\sum_{\left(i,j\right)}D_{ij}}$$ (37)

where $D_{ij}$ is the weight of the edge connecting nodes i and j.

The enhanced Newman fast partitioning algorithm advances the conventional approach by integrating multiple correlation indicators into the TOPSIS framework with entropy-based weighting, thereby replacing the reliance on a single correlation measure and facilitating a more objective allocation of weights.

### 4.3 Dynamic subdivision process

**Fig 7:**
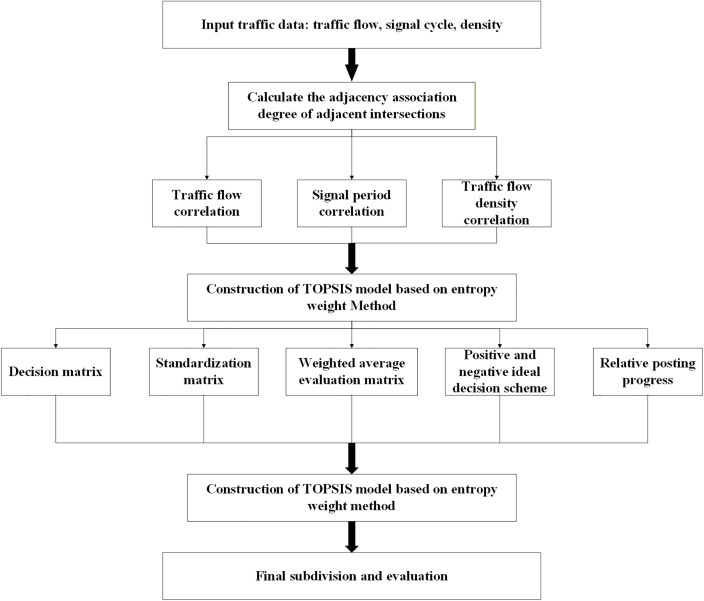
Dynamic subdivision process flowchart.

An accurate and precise partitioning of the transportation network is essential for the effective governance of sub-regions. The traffic network, characterized as a complex system, is segmented into control sub-regions through an enhanced Newman fast division algorithm that utilizes a measure of relative closeness as the edge weight. This approach ensures that the resulting sub-regions more accurately represent the operational characteristics of the road network. The detailed division procedure, as illustrated in Fig 7, comprises several sequential steps.

1) Step 1. Represent intersections as nodes and road segments connecting them as edges. Assign a unique label to each node.
2) Step 2. Utilize traffic flow data, including metrics such as traffic volume and signal timings, collected through traffic detection devices, along with fundamental road network information, to compute the correlation between adjacent intersections using the correlation degree model.
3) Step 3. Input the computed correlation coefficient between adjacent intersections into the TOPSIS algorithm employing the entropy weight method. The entropy weight method is used to determine the weights of the indices, while TOPSIS calculates the relative closeness degree. Finally, construct the edge weight matrix utilizing the obtained relative closeness degrees.
4) Step 4. Utilize intersections as nodes and their spatial proximity as edge weights, subsequently inputting this data into the advanced Newman fast partitioning algorithm. Calculate the modularity, iteratively merge nodes to optimize it, and select the partition that maximizes modularity as the basis for traffic control.

## 5 Model verification

**Fig 8:**
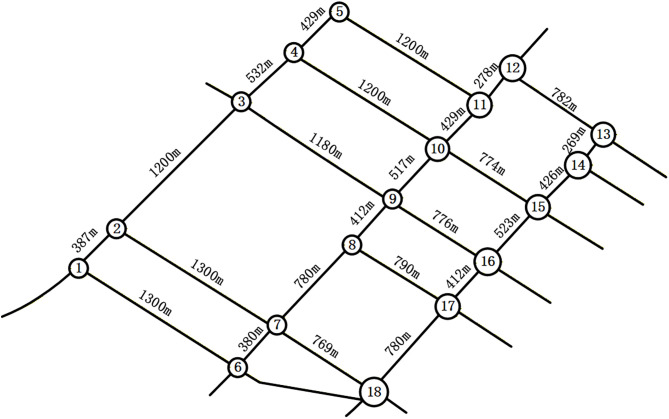
Simplified map of the regional road network for sub-region division.

Model validation was conducted using traffic flow data from the Changchun Traffic Management Platform. The study network comprises 18 signalized intersections and 38 segments, featuring grid and radial configurations with significant spatiotemporal heterogeneity and tidal fluctuations. For analysis, the network was abstracted into a topological graph where signalized intersections are represented as nodes and road segments as edges, as illustrated in Fig 8.

### 5.1 Correlation degree calculation

Utilizing open network data and Autonavi Map, information such as the distances between adjacent intersections and the number of lanes is systematically collected for validation purposes, as detailed in Table 4. Publicly available data are also analyzed to determine the cycle durations and traffic volumes at intersections during the period from 17:00–18:00 on a specific day within the region, along with the distances between successive intersections, as depicted in Table 5. The correlations among intersections are quantified using a correlation degree model, and the corresponding weight coefficients are calculated via the entropy weight method implemented in MATLAB. The resulting correlation degree values are compiled in Table 6.

**Table 4:** Correlation degree related calculation data.

Intersection	V (vch/h)	C(s)	N (bar)
1	2200	114	3
2	2827	132	4
3	2235	114	4
4	3236	142	4
5	3081	147	4
6	3212	132	3
7	3158	157	4
8	2313	113	3
9	2396	124	4
10	3698	152	5
11	3584	146	3
12	2323	114	2
13	2173	109	2
14	2823	136	4
15	3514	142	4
16	1949	119	2
17	2345	129	3
18	3059	172	4

**Table 5:** Spacing between adjacent intersections.

i−j	Distance	i−j	Distance
1−2	387	8−17	790
1−6	1300	9−10	517
2−3	1200	9−16	776
2−7	1300	10−11	429
3−4	532	10−15	774
3−9	1180	11−12	278
4−5	429	12−13	782
4−10	1200	13−14	269
5−11	1200	14−15	426
6−7	380	15−16	523
7−8	780	16−17	412
7−18	769	17−18	780
8−9	412		

**Table 6:** The degree of correlation between adjacent intersections.

i↔j	$I_{ij}$	i↔j	$I_{ij}$
1↔2	0.49	8↔17	0.88
1↔6	0.89	9↔10	1.10
2↔3	0.61	9↔16	0.63
2↔7	1.53	10↔11	1.41
3↔4	0.89	10↔15	0.84
3↔9	0.51	11↔12	0.47
4↔5	0.85	12↔13	0.36
4↔10	1.06	13↔14	0.42
5↔11	0.85	14↔15	0.58
6↔7	0.79	15↔16	0.77
7↔8	0.60	16↔17	0.50
7↔18	0.96	17↔18	0.38
8↔9	0.34		

### 5.2 Intersection relative proximity value

**Fig 9:**
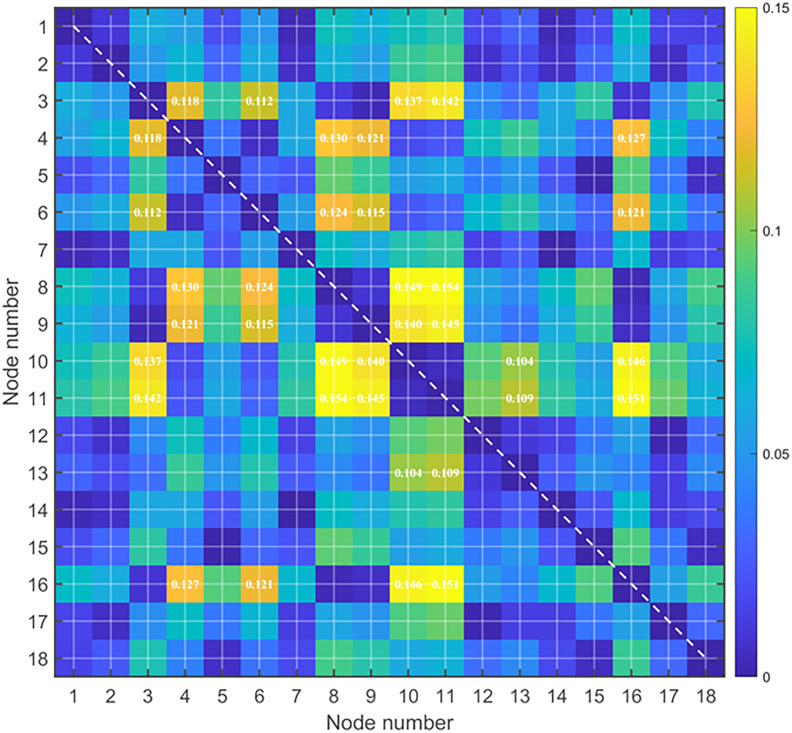
Heat map of node relative closeness.

Relative closeness is determined by calculating Euclidean distances to positive and negative ideal solutions, with node-specific results detailed in Table 7. These values function as edge weights in both the standard and enhanced Newman fast algorithms for sub-region partitioning. The heatmap in Fig 9 visualizes the spatial distribution of node performance relative to the network average of 0.52. In this representation, cooler colors indicate higher closeness with minimal variance, while warmer colors denote higher closeness with significant disparity. Specifically, Node 11 achieves the maximum closeness value of 0.592, identifying it as a pivotal junction with the strongest overall correlation in the network.

**Table 7:** Node relative closeness.

Intersection	$D_{i}$	Intersection	$D_{i}$
1	0.512	10	0.587
2	0.502	11	0.592
3	0.450	12	0.494
4	0.568	13	0.483
5	0.533	14	0.509
6	0.562	15	0.532
7	0.509	16	0.441
8	0.438	17	0.496
9	0.447	18	0.527

### 5.3 Results of traffic control sub-region division

**Fig 10:**
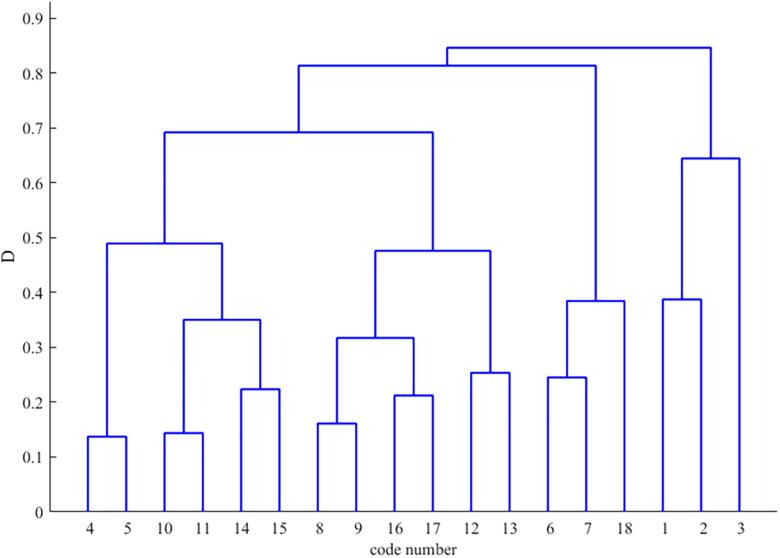
Tree diagram of partitioning results for Newman’s fast partitioning algorithm.

**Fig 11:**
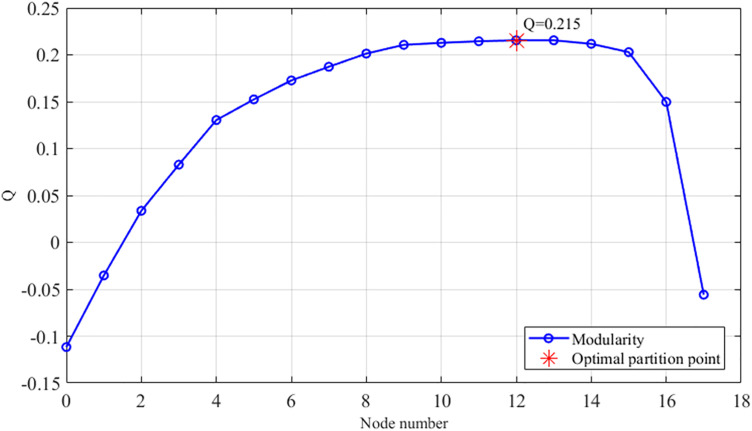
Newman's fast division algorithm divides modularity.

To validate the proposed methodology, regional roadway networks were partitioned using the Newman fast division algorithm and its enhanced variant. This process utilized an intersection correlation degree model as input within a MATLAB environment. As shown in Fig 10, the traditional Newman algorithm divides the road network into four distinct control sub-regions. Sub-region 1 includes intersections 1, 2, and 3. Sub-region 2 encompasses intersections 4, 5, 10, 11, 14, and 15. Sub-region 3 consists of intersections 6, 7, and 18, while sub-region 4 contains intersections 8, 9, 12, 13, 16, and 17. The modularity values generated during this merging process are detailed in Fig 11, where the optimal partition occurs near node 12 with a modularity value of 0.215.

**Fig 12:**
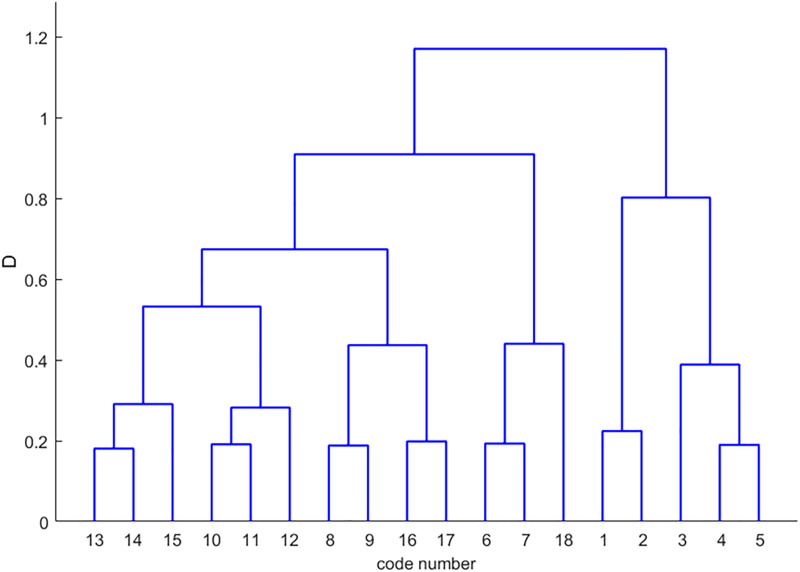
Tree diagram of partitioning results for improved Newman’s fast partitioning algorithm.

**Fig 13:**
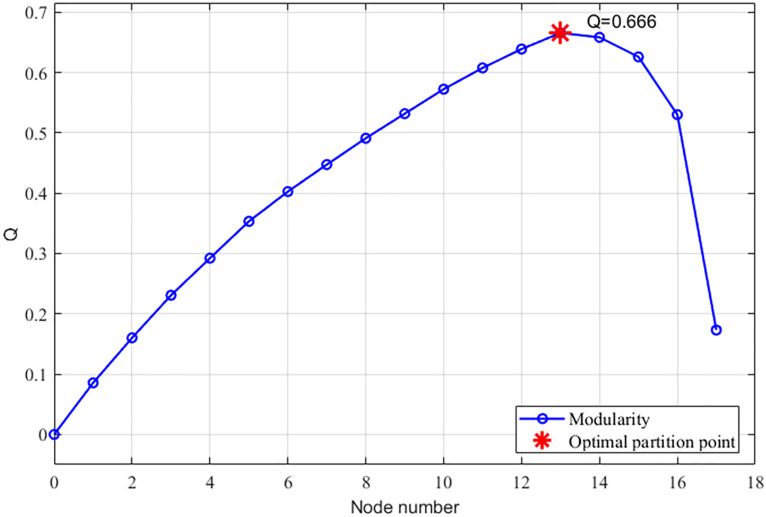
Improve the modularity of the Newman algorithm.

The study further introduces an improved Newman algorithm that incorporates an entropy weight-based TOPSIS model to calculate dynamic edge weights. These weights integrate multi-dimensional traffic features such as volume, signal timing, and density. The resulting network segmentation is illustrated in Fig 12, which also defines four sub-regions. In this refined model, sub-region 1 comprises intersections 1–4, sub-region 2 contains intersections 6, 7, and 18, sub-region 3 includes intersections 8, 9, 16, and 13, and sub-region 4 consists of intersections 10–15. The corresponding modularity variation curves are provided in Fig 13.

Analysis of the modularity metrics reveals that the enhanced algorithm achieves a maximum value of 0.666, representing a substantial improvement over the traditional value of 0.215. This absolute increase of 0.451 reflects a relative enhancement of nearly 210 percent, indicating a superior structural characterization of the traffic network. From a traffic engineering perspective, higher modularity signifies that sub-regions possess greater internal cohesion and fewer inter-regional connections. High internal homogeneity is vital for green-wave progression, ensuring sub-regions maintain correlated traffic rhythms. This alignment optimizes phase synchronization and reduces boundary delays by preventing control unit fragmentation during transient peak periods.

**Fig 14:**
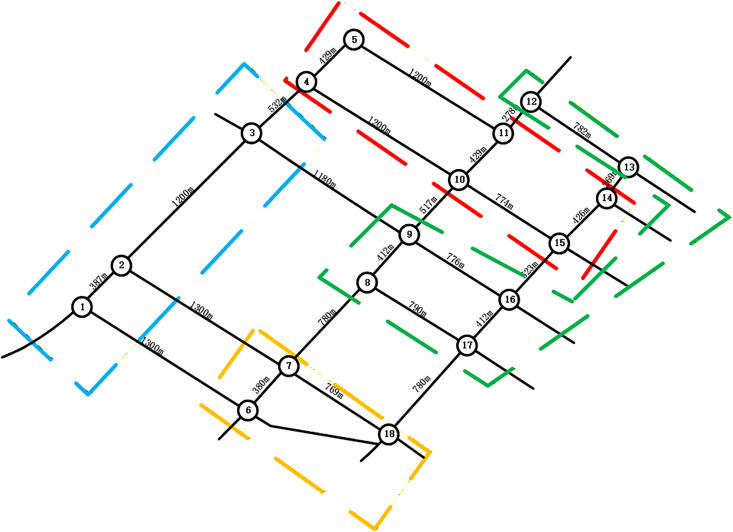
The Newman fast partitioning algorithm controls the results of sub-region partitioning.

**Fig 15:**
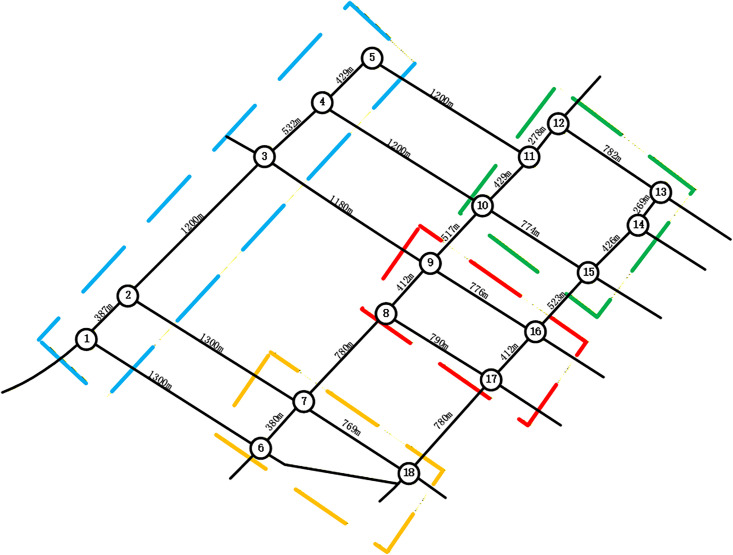
Improve the Newman fast partitioning algorithm to control the results of sub-region partitioning.

Furthermore, the integration of dynamic edge weights allows the algorithm to maintain robust interactions between nodes, preventing the artificial segmentation often seen in purely topological approaches. A final comparative analysis of the division outcomes is presented in Figs 14 and 15. These results demonstrate that the improved algorithm provides higher granularity by effectively delineating small-scale control units that capture local traffic similarity patterns. By subdividing large, heterogeneous clusters into more precise units—such as the allocation of nodes 10, 11, 14, and 15 to a specific sub-region—the methodology facilitates more responsive and decentralized traffic management in complex urban environments.

An analysis of the traffic control zones generated by both algorithms reveals that the enhanced Newman fast division algorithm yields more detailed and realistic results. The proposed algorithm optimizes network structure and alleviates congestion. Enhanced modularity fosters sub-region stability, while partitioning at minimal-correlation boundaries ensures consistent signal control and mitigates performance losses from signal offsets.

### 5.4 Evaluation of the division results of traffic control sub-regions

Various metrics are employed to evaluate sub-region segmentation algorithms, including modularity, computational complexity (both temporal and spatial), and benchmarking against graph datasets. Nonetheless, these metrics exhibit several limitations, such as limited adaptability and dependence on single evaluation criteria. In contrast, metrics such as division density and normalized mutual information (NMI) demonstrate greater flexibility and rigor. Given the absence of an unequivocal “true” traffic subdivision, a benchmark partition derived from multi-source information fusion was utilized as an NMI reference. This benchmark integrates link saturation clustering during peak traffic hours, road network connectivity, and traffic management insights. Comparing algorithm outputs with this benchmark allows NMI to serve as an effective measure of practical accuracy.

To distinguish between the results of the Newman Fast and the enhanced Newman Fast partitioning algorithms, this study utilizes normalized mutual information (NMI) and partition density as quantitative evaluation metrics.

#### 5.4.1 Normalization information.

Normalized Mutual Information (NMI [39]) serves as an entropy-based metric for assessing the similarity between the outputs of algorithms and true sub-regions. Elevated entropy values correspond to increased fragmentation, with the metric ranging from 0 to 1. Values approaching 1 denote a strong correspondence with actual sub-regions, as delineated in Formula.


$$NMI\left(Y,C\right)=\frac{2\times I(Y;C)}{H\left(Y\right)+H\left(C\right)}$$ (38)

where Y,C represents the sub-region label output by the algorithm and the real sub-region label. I(Y;C) represents mutual information, and Y and C measure the degree of information overlap. H(Y) and H(C) are the entropies of two label sets.

#### 5.4.2 Division density.

Partition density PD [40] quantifies the edge density within a sub-region, functioning as a weighted average of the edge densities across individual sub-regions. It serves as a metric for assessing sub-region quality: High PD indicates strong internal links, supporting efficient signal control like green waves, and sparse external connections, enabling independent optimization and simpler, more robust systems. Higher PD reflects dividing areas with high internal cohesion and low external coupling, aligning with traffic control goals. The corresponding formula is presented in (49).


$$PD=\frac{\text{2}}{m}\sum_{c}m_{c}\cdot \frac{m_{c}-(n_{c}-1)}{\left(n_{c}-2)(n_{c}-1\right)}$$ (39)

The number of edges and nodes within the network and its sub-regions is a significant parameter. Partition density (PD) serves as a metric to evaluate the quality of the partitioning, where a higher PD reflects stronger internal connectivity, thereby facilitating efficient signal management such as green wave coordination. Additionally, a higher PD indicates fewer interconnections between sub-regions, which diminishes traffic flow coupling and promotes autonomous control strategies. Increased partition density minimizes inter-zonal coupling, facilitating independent signal optimization and preventing spatial delay propagation. This approach offers a decentralized management framework that effectively balances local control with network-wide stability.

#### 5.4.3 Evaluation and analysis.

**Fig 16:**
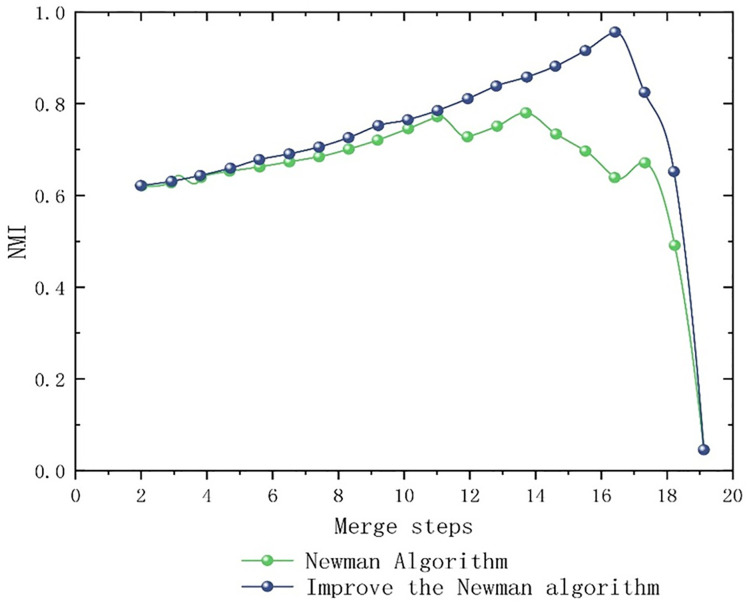
NMI contrast figure.

This study utilizes normalized mutual information (NMI) and partition density (PD) as metrics for evaluating partitioning results. NMI measures the similarity between algorithm-generated partitions and the true sub-region structure, as depicted in Fig 16. Upon the inclusion of node 16, the optimized Newman fast partitioning algorithm attains its maximum performance relative to the standard implementation. Prior to the addition of node 16, the enhanced algorithm more accurately corresponds to the actual sub-region partitioning than the original Newman algorithm, thereby demonstrating its superior efficacy.

**Fig 17:**
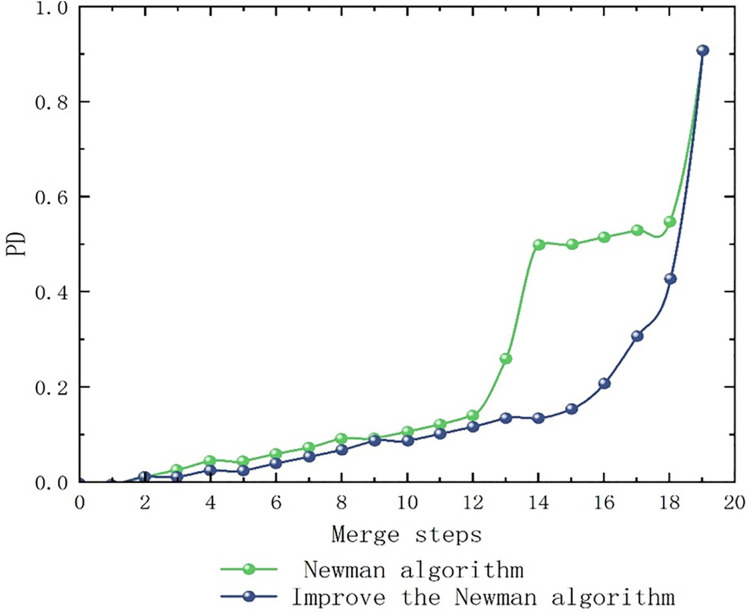
PD contrast figure.

Secondly, the division density PD evaluates the quality of sub-regional outcomes through the analysis of internal edges and connections. As depicted in Fig 17, following the merging to node 16, the improved Newman Fast Division algorithm attained its peak performance relative to the original algorithm. Prior to reaching node 16, it demonstrated consistent superiority over the original, thus indicating enhanced community detection capabilities. Consequently, the refined algorithm exhibits increased effectiveness.

By comparing the Newman Fast partitioning algorithm with its enhanced variant, which incorporates normalized mutual information (NMI) and partitioning density (PD) indices, it becomes apparent from the elevated NMI and PD values displayed in Figs 16 and 17 that the improved algorithm offers a more precise and higher-quality delineation of sub-region structures than the original Newman Fast algorithm. This refined delineation aligns more accurately with the actual boundaries of sub-regions and more effectively identifies high-quality community structures. These results confirm that superior partition density significantly reduces inter-regional coordination complexity. Such improvements foster stable, autonomous traffic management, allowing sub-regions to maintain optimal cycle lengths with minimal external interference.

### 5.5 Algorithm comparison analysis

To validate the efficacy of the enhanced Newman fast division algorithm, a comparative analysis was conducted involving four benchmark algorithms: an association-enhanced Louvain method, label propagation, spectral clustering utilizing boundary metrics, and Spinglass. These algorithms were evaluated under identical data and environmental conditions within a sub-region of a road network, with performance assessed through the Normalized Mutual Information (NMI) and Partition Density (PD) metrics. The findings are summarized in the accompanying Table 8.

**Table 8:** Performance comparison of different partitioning algorithms.

Algorithm	Q	NMI	PD
Newman	0.215	0.500	0.30
Spinglass	0.382	0.850	0.750
Improved Louvain	0.450	0.813	0.722
Improved Newman	0.666	0.970	0.90
LPA	0.5526	0.797	0.450

Table 8 presents a comparative analysis of the enhanced Newman fast division algorithm against baseline methodologies—including the traditional Newman algorithm, the improved Louvain method, the Label Propagation Algorithm, and spectral clustering—evaluated using metrics such as modularity Q, normalized mutual information (NMI), and partition density (PD). All techniques employ identical edge weights derived from entropy weight-TOPSIS, ensuring that observed differences in partition quality are attributable solely to the underlying algorithms.

– illustrate that the improved Newman Fast Algorithm outperforms the baseline in metrics such as modularity, NMI, and PD, achieving a maximum Q value of 0.666 in comparison to 0.215. This substantiates the benefits of employing traffic-aware weighted edges.

**Fig 18:**
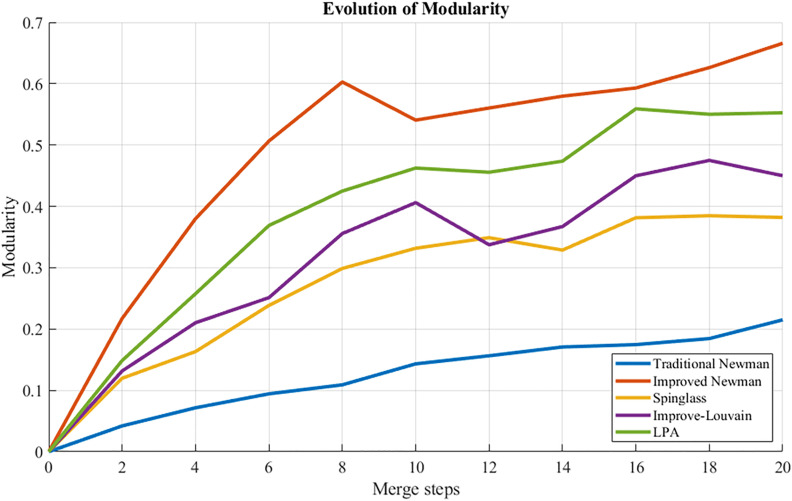
Modularity comparison: curves of different algorithms.

**Fig 19:**
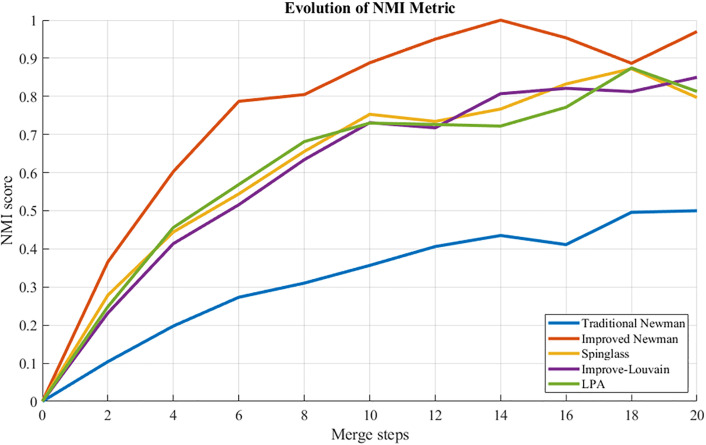
NMI comparison: curves of different algorithms.

**Fig 20:**
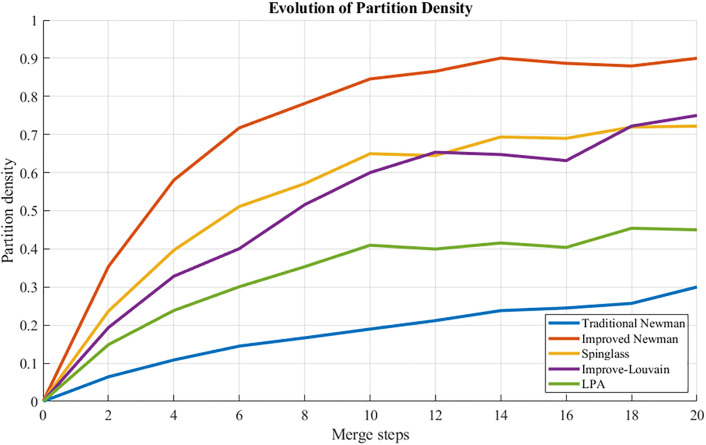
PD comparison: curves of different algorithms.

**Fig 21:**
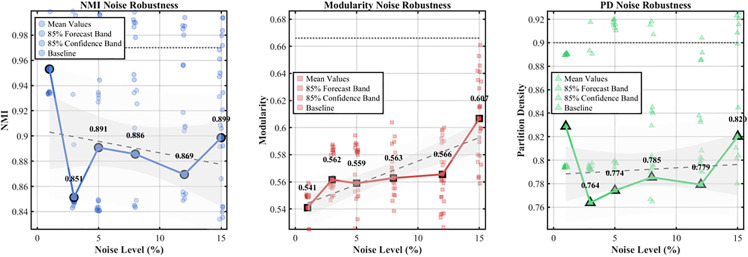
Enhancing the noise robustness testing of the Newman algorithm.

To evaluate the robustness of the methodology, Gaussian noise with a variance of ±5% was introduced to the input traffic features. Subsequent experiments were conducted, as depicted in Fig 21. The findings suggest that the degradation in performance of the improved Newman algorithm, measured by NMI, PD, and modularity, is less significant than that observed in the baseline, thus indicating its superior stability under measurement noise conditions.

## 6 Conclusion

This paper presents a systematic methodology for partitioning traffic control sub-regions is presented to enhance urban arterial efficiency and reflect real-world flow patterns. This dynamic division technique integrates an advanced Newman fast partitioning algorithm with a multi-factor correlation model. By analyzing volume, signal timing, and density at adjacent nodes, a correlation framework utilizing entropy-weighted TOPSIS determines the relative closeness between intersections. These closeness values serve as edge weights for the community detection process.

Empirical results confirm superior accuracy in sub-region delineation and higher consistency with actual traffic conditions. Specifically, the enhanced Newman algorithm achieves a maximum modularity of 0.666, representing a significant improvement over the 0.215 benchmark obtained through traditional methods. This absolute increase of 0.451 validates the effectiveness of the integrated modeling strategy.

While the enhanced Newman algorithm demonstrates superior sub-region partitioning, future research will assess its scalability in larger urban networks and integrate more complex dependencies into the correlation model. Subsequent validation via VISSIM or SUMO simulations will facilitate rigorous comparative analyses against conventional methods, evaluating performance through metrics such as vehicle delay and throughput. These efforts will further substantiate the algorithm's practical efficacy and robustness in real-world urban traffic management.

Future research addresses current limitations by incorporating spatiotemporal dependencies among non-adjacent nodes to refine macro-level traffic pattern representations. Algorithm optimization involves investigating approximate methods and distributed computing frameworks to mitigate computational overhead in extensive networks. Comprehensive empirical validation on diverse, large-scale urban infrastructures ensures metropolitan-scale utility and real-world applicability.

## Supporting information

S1 Data Traffic indicators and algorithm performance evaluation data. This file contains the raw traffic indicators for various intersections, modularity (Q) comparison results, and performance evaluation metrics including NMI and PD.
(XLSX)

S2 Data Calculation procedures and results of the Entropy Weight-TOPSIS model. This file provides the details of weight allocation, normalization matrices, and edge weight calculation results.
(XLSX)

S3 Data Source data for traffic parameter visualizations. This file includes the numerical datasets used to generate the waterfall charts for traffic volume, signal cycle, and traffic flow density.
(XLSX)

S1 Code MATLAB source code for the proposed algorithm and analysis. This compressed archive contains the complete MATLAB scripts used to implement the improved Newman fast partitioning algorithm.
(ZIP)
