# Supplementary material for: An improved Newman fast division algorithm based on multi-factor correlation for dynamic traffic sub-region control
Source: PLoS One. 2026 Mar 12;21(3):e0343245. doi: 10.1371/journal.pone.0343245 (PMC12981514; doi:10.1371/journal.pone.0343245)
Supplement: S1 Code — This compressed archive contains the complete MATLAB scripts used to implement the improved Newman fast partitioning algorithm. (ZIP) [file pone.0343245.s004.zip › S4_Code/data/Report on the Calculation Process of Entropy-Weighted TOPSIS.pdf]

Entropy-Weighted TOPSIS Calculation Report

Generation Timestamp: December 6, 2025, 12:43:16

1. Data Overview

Sample Size: 25 pairs of adjacent intersections.

Evaluation Indicators: Three correlation degree indicators.

Data Source: Correlation data derived from Table 3.

2. Entropy Weight Assignment Results

The weights assigned to each indicator based on the entropy method are as follows:

Traffic Operation Correlation: 28.35%

Signal Coordination Correlation: 47.46%

Traffic Status Correlation: 24.19%

Dominant Indicator: Traffic Operation Correlation

3. TOPSIS Evaluation Results

Maximum Relative Closeness : 1.0000 (Pair 2-7)

Minimum Relative Closeness : 0.0727 (Pair 12-13)

Average Relative Closeness: 0.3531

4. Output Files

Primary Output File: Entropy_Weighted_TOPSIS_Calculation_Process.xlsx

Included Sheets/Tables:

Decision Matrix (Raw Data Table)

Normalized Decision Matrix (Intermediate Table)

Feature Proportion Matrix (Entropy Calculation)

Detailed Entropy Calculation Process

Final Weight Assignment Table

TOPSIS Evaluation Results

TOPSIS Ranking Results

Summary of Calculation Parameters
