# Supplementary material for: An improved Newman fast division algorithm based on multi-factor correlation for dynamic traffic sub-region control
Source: PLoS One. 2026 Mar 12;21(3):e0343245. doi: 10.1371/journal.pone.0343245 (PMC12981514; doi:10.1371/journal.pone.0343245)
Supplement: S1 Code — This compressed archive contains the complete MATLAB scripts used to implement the improved Newman fast partitioning algorithm. (ZIP) [file pone.0343245.s004.zip › S4_Code/README_Code_Description.pdf]

Manuscript ID: PONE-D-25-51433R2 Title: An improved Newman fast division algorithm based on multi-factor correlation for dynamic traffic sub-region control File: S4_Code.zip (Supporting Information)

README: Description of Code and Data Files

1. Overview

This archive contains the source code (MATLAB) and dataset used to generate the results, figures, and tables presented in the manuscript. The algorithms implemented include the proposed "Improved Newman Fast Partitioning Algorithm based on Multi-factor Correlation" and several benchmark algorithms (Traditional Newman, Spinglass, Louvain, LPA) for comparison.

2. System Requirements

- Software: MATLAB (Recommended version: R2020b or later).
- Toolboxes: Statistics and Machine Learning Toolbox (required for functions such as kmeans, linkage, zscore, etc.).

3. File Manifest & Description

A. Main Execution Script

- Main.m: This is the primary entry point for the simulation. It integrates all parts of the experiment, including:
    - Data preparation and initialization.
    - Calculation of the Improved Correlation Model.
    - Entropy Weight-TOPSIS calculation for relative closeness.
    - Comparison of five community detection algorithms.
    - Noise robustness testing (NMI, Modularity, Partition Density).
    - Generation of performance retention analysis graphs.

B. Algorithm Implementations

- Improve_Newman_Fast_Partitioning_Algorithm.m: The core implementation of the proposed method. It utilizes the multi-factor correlation and relative closeness to construct edge weights and performs the improved community detection.
- Newman_Fast_Partitioning_Algorithm.m: Implementation of the traditional Newman

fast algorithm for baseline comparison.

- Comparative_Test.m: A dedicated script for running comparative experiments between different algorithms and performing extensive noise robustness analysis.
- EntropyWeight_TOPSIS_Calculation.m: Performs the specific Entropy Weight and TOPSIS calculations to determine node weights and relative closeness based on traffic data (Table 3 in the manuscript). It generates the Excel file EntropyWeight_TOPSIS_Calculation_Tables.xlsx containing detailed intermediate steps.

C. Metric Analysis & Visualization

- NMI.m: Script for calculating and visualizing the Normalized Mutual Information (NMI) evolution during the community merging process. Compares the original and improved algorithms.
- PD.m: Script for calculating and visualizing the Partition Density (PD) evolution.
- synthesize_Traffic_Waterfall.m: Generates synthetic traffic data and creates the 3D waterfall plots (traffic volume, signal period, density) used to visualize traffic parameter relationships.

D. Data Files

- data/: Folder containing auxiliary data files (if applicable).
- Various .m helper functions are included within the main scripts or as standalone files to support calculations (e.g., calculate_nmi, calculate_PD).

4. Usage Instructions

1. Unzip the S4_Code.zip archive to a local folder.
2. Open MATLAB and set the Current Folder to the unzipped directory.
3. To reproduce the full experimental results:
    - Run Main.m.
    - The script will output numerical results to the Command Window and generate all relevant figures (Clustering Dendrograms, Robustness Curves, Heatmaps, etc.).
4. To view specific calculation details:
    - Run EntropyWeight_TOPSIS_Calculation.m.

- This will generate an Excel file named EntropyWeight_TOPSIS_Calculation_Tables.xlsx in the root directory, showing the step-by-step weight calculation process.

5. Output Files

Upon execution, the code may generate the following files in the working directory:

- EntropyWeight_TOPSIS_Calculation_Tables.xlsx: Detailed calculation tables.
- Noise_Robustness_Combined.png: Visualization of robustness tests.
- Performance_Retention_Analysis.png: Performance retention rates under noise.
- traffic_waterfall_data.mat: Generated traffic simulation data.
